# Identification and characterization of pathogenicity-related genes of *Rhizoctonia solani* AG3 during tobacco infection

**DOI:** 10.3389/fpls.2022.1116506

**Published:** 2023-01-17

**Authors:** Qianjun Tang, Qianqian Ma, Zhipeng Xiao, Yansong Xiao, Yunsheng Wang, Lei Liu, Weiye Peng, Bing Wang, Tianbo Liu, Na Song

**Affiliations:** ^1^ College of Plant Protection, Hunan Agricultural University, Changsha, Hunan, China; ^2^ Tobacco Research Institute of Hunan Provence, Changsha, Hunan, China

**Keywords:** rhizoctonia solani, tobacco, pectin, P450 genes, target spot disease

## Abstract

Tobacco target spot disease is caused by a ubiquitous soil-borne phytopathogen *Rhizoctonia solani*; the pathogenic mechanisms underlying the effects of *R. solani* remain unclear. Deeper understanding of the functional responses to *R. solani* during host plant infection would help identify the molecular mechanisms essential for successful host invasion. In this study, we performed global transcriptional analysis of *R. solani* during various stages (12, 24, 48, 72, 96, and 120 h) of tobacco infection *via* an RNA sequencing method, while utilizing the pathosystem model *R. solani* AG3–tobacco (*Nicotiana tabacum* L.). After *R. solani* inoculation, the number of differentially expressed genes of *R. solani* differed at the various time points. Moreover, several gene ontology and Kyoto encyclopedia of genes and genomes pathways were unique in different infection stages, especially with respect to the genes involved in plant cell wall degradation and catalysis of biotransformation reactions, such as the pectin metabolic process and pectin catabolic process. The overexpressing-PD8 *N. benthamiana* plants enhanced the susceptibility to *R. solani.* In addition, we found that large amounts of reactive oxygen species (ROS) were generated in tobacco after infected by *R. solani*. *R. solani* encoding *FAD/NAD binding oxidoreductase* and *peroxidase gene* family to eliminating ROS and counteract oxidative stress. Moreover, *Perox3* was validated that can enhance the ability of scavenging ROS by co-injecting. Overall, our findings show that pectin-degrading enzymes and cytochrome P450 genes are critical for plant infection. These results provide comprehensive insights into *R. solani* AG3 transcriptome responses during tobacco invasion.

## 1 Introduction

Target spot disease was first detected in Brazil in 1984 on tobacco plants (*Nicotiana tabacum* L.) and subsequently spread widely worldwide, including in Costa Rica, USA, and China (Ahsan et al., 2017). The classic symptoms of target spot disease are circular spots with concentric rings on tobacco leaves (Sun et al., 2022). The disease commonly occurs during the growth and maturity periods of the plant, with short latency and high transmissibility, resulting in huge economic losses due to decreased tobacco yield and quality. Tobacco target spot is caused by *Rhizoctonia solani* Kühn (sexual form, *Thanatephorus cucumeris* (Frank) Donk), a ubiquitous soil-borne phytopathogen that infects various important crops such as rice (sheath blight disease [SBD]), corn (rhizoctonia root rot disease), potatoes (black scurf disease), soybean (rhizoctonia foliar blight disease), tomato (tomato rhizoctonia rot disease), cabbage (head rot disease), and lettuce (bottom rot disease) (Lin et al., 2021).

*R. solani* is classified into 14 distinct anastomosis groups (AG-1 to AG-13 and AGBI) on the basis of presence or absence of hyphal fusion; distinct groups infect different host plants (Abdoulaye et al., 2019). Generally, AG1-IA primarily infects rice plants, AG2 infects cruciferous vegetables, and AG3 prefers solanaceous plants under natural conditions (Hane et al., 2014). Rice SBD caused by AG1-IA and potato black scurf disease caused by AG3 have been widely investigated because of their economic and ecological importance in agriculture (Zrenner et al., 2020; Yang et al., 2022). However, relatively few studies have examined the relationship between *R. solani* AG3 and other economically important crops, including tobacco. Recently, the draft genome sequence of *R. solani* AG3 became publicly available and was refined, thereby providing thorough knowledge of the architecture, variation, and evolution of the pathogen and enabling more in-depth genetic study of *R. solani* AG3 (Shu et al., 2021; Kaushik et al., 2022).

High-throughput omics technology provides deeper insights into biological system responses to complex perturbations and is an attractive tool for plant–pathogen interaction research (Peng et al., 2021). RNA sequencing (RNA-seq) is a proximity bias–free measure that can be used to objectively and exhaustively investigate fluctuations in the transcript abundance of an organism in a more precise manner than oligonucleotide array hybridization (Zhang, 2021). Recently, the *R. solani* infection mechanism and host defense mechanisms were examined at the genomic and transcriptomic levels. These studies included the following: *R. solani* AG1-IA, which causes SBD in rice (Wang et al., 2022), banded leaf and sheath blight in maize (Cao et al., 2021), and foliar blight in soybean (Yamamoto et al., 2019); *R. solani* AG1-IB, which causes bottom rot in lettuce (Verwaaijen et al., 2017); and several other pathosystems. In a previous transcriptomic study that involved hyphal growth on media and also studied the interaction between *R. solani* AG3-PT and potato sprouts at the early and late infection stages, peptidase-encoding genes showed strong differential expression at an early infection stage, and hydrolase-encoding genes targeting cell wall components were highly induced at the late infection stage (Zrenner et al., 2021). To date, very few studies have used genomic analysis to examine the global transcript levels in response to *R. solani* AG3 infection of tobacco plants.

In this study, the transcriptomes of tobacco inoculated with *R. solani* AG3 were analyzed at different time points (12, 24, 48, 72, 96, and 120 h) by using RNA-seq technology. Several gene ontology (GO) and Kyoto encyclopedia of genes and genomes (KEGG) pathways were found to be unique in different infection stages, especially with respect to the genes involved in plant cell wall degradation and catalysis of biotransformation reactions. Apart from that, we demonstrated that generation of the ROS is a major defense mechanism of tobacco against the *R. solani* and *R. solani FNBO* and *Perox3* gene family detoxify the harmful effects of ROS generated from tobacco defense responses.The RNA-seq data generated provide valuable information for understanding the *R. solani* AG3 response during tobacco infection and could be useful for future studies on the mechanisms underlying the plant’s defense response.

## 2 Materials and methods

### 2.1 Sample collection and *R. solani* inoculation

Tobacco plant (K326 cultivar) and *R. solani* AG3 were used in this study. All plants were grown in the greenhouse at 25°C and 70% relative humidity with a 12/12h of day/night light. The fifth and sixth leaves from the lower part of a nine-leaf–stage potted tobacco plant were collected for friction inoculation. A mycelium-containing agar disk (diameter, 5 mm) was inoculated at the inoculation point, with a friction area of approximately 4 mm^2^. The mycelial plug was inoculated on the tobacco leaf according to the above-mentioned method for the control; six inoculation points were selected on each tobacco leaf. The plant was grown in a greenhouse at 25°C and 90% relative humidity. The fungal cake was removed 2 d after inoculation. The infection status was analyzed at the inoculation points after 6, 12, 24, 48, 72, 96, and 120 h. Symptomatic leaves were obtained from the infected symptomatic tobacco plants. Samples from each of the three tobacco plants were harvested during each treatment and pooled as a mixed line. Samples were stored at -80°C until use for RNA-seq. In total, 21 samples were analyzed: seven time points × three replicates each.

### 2.2 RNA extraction and RNA-seq library preparation

Total RNA was extracted as previously described (Peng et al., 2021). Briefly, approximately 0.1 g of symptomatic leaves per sample was ground into a homogenate in liquid nitrogen, and 1 mL of TRIzol (TransGen, Beijing, China) was added to each sample. The total RNA–containing supernatant was washed with an equivalent volume of chloroform; the total RNA was precipitated using isopropanol and dissolved in RNase-free water with RNase inhibitors. The total RNA quality and quantity were assessed using a SpectraMax NanoDrop system (Thermo Fisher Scientific, MA, USA). All samples had RNA integrity numbers higher than 8.0, indicating relatively intact and protein-free RNA. The RNA-seq library was prepared using 4 μg total RNA, with the KAPA Stranded mRNA-Seq Library Preparation Kit (Kapa Biosystems, Roche, Basel, Switzerland). RNA-seq libraries were used for cluster formation with the HiSeq X PE Cluster Kit V2.5 on an Illumina cBOT cluster generation system (Illumina) and sequenced in one lane by using the Illumina HiSeq 2500 platform in the 100 bp paired-end mode.

### 2.3 Bioinformatics analysis

Raw data were separated by barcodes by using the Illumina pipeline (CASAVA, version 1.8.2). Illumina adapters were removed using the Cutadapt software (version 1.4.1), and base calls with average phred scores of less than 20 and lengths below 50 bp were filtered out using the Trimmomatic software (version 0.39). The filtered and trimmed reads in each library were aligned by using TopHat2 version 2.1.0. GO enrichment was functionally annotated using Blast2GO version 2.5 against the non-redundant protein sequence database of the National Center for Biotechnology Information (NCBI). Differentially expressed genes (DEGs) were identified by mapping each gene to the KEGG database for pathway enrichment by using clusterProfiler3 version 4.0. KEGG pathway enrichment analysis of the correlation was performed using p-values adjusted using Benjamini–Hochberg correction for the false discovery rate.

### 2.4 Detection of ROS accumulation

To observe ROS accumulation, the tobacco leaves were harvested at 72 hours after inoculated with *R. solani* AG3. The harvested tobacco leaves were stained with (2 mg/ml, pH 4) 3,3’-Diaminobenzidine (DAB) in the dark overnight, then add decolorizing solution (ethanol/acetic acid/glycerol 3:1:1) to decolorize the leaves at 95°C for 15 min. To evaluate the elimination effect of ROS, the pYBA1143-based constructs of *Perox* genes were transformed into the Agrobacterium tumefaciens strain EHA105 and co-injected with apoptosis regulator BAX. The BAX and pYBA1143 vector served as a positive and negative control, respectively.

### 2.5 qPCR validation of differential expression data

Samples were collected under identical conditions and at similar time points for real-time PCR (qPCR) analysis. The experiment was performed in triplicates and repeating three times. Reverse transcription and qPCR assays were performed as previously described (Peng et al., 2022), with minor modifications. RNA (1 μg) was reverse-transcribed to cDNA by using HiScript III All-in-one RT SuperMix Perfect (Vazyme, Nanjing, China), following the manufacturer’s protocols. All the primers were designed using the online GenScript Real-time PCR Primer Design software and checked for specificity by using the NCBI Primer-BLAST bioinformatics tool. Primers are provided in **Table S2**. qPCR was performed using NovoStart SYBR qPCR SuperMix Plus (Novoprotein, Suzhou, China), according to the manufacturer’s protocol, on a C1000 Touch Thermal Cycler with a CFX96 Real-Time System (Bio-Rad, CA, USA).

## 3 Results

### 3.1 Tobacco leaf symptoms after *R. solani* AG3 inoculation

**Figure 1:**
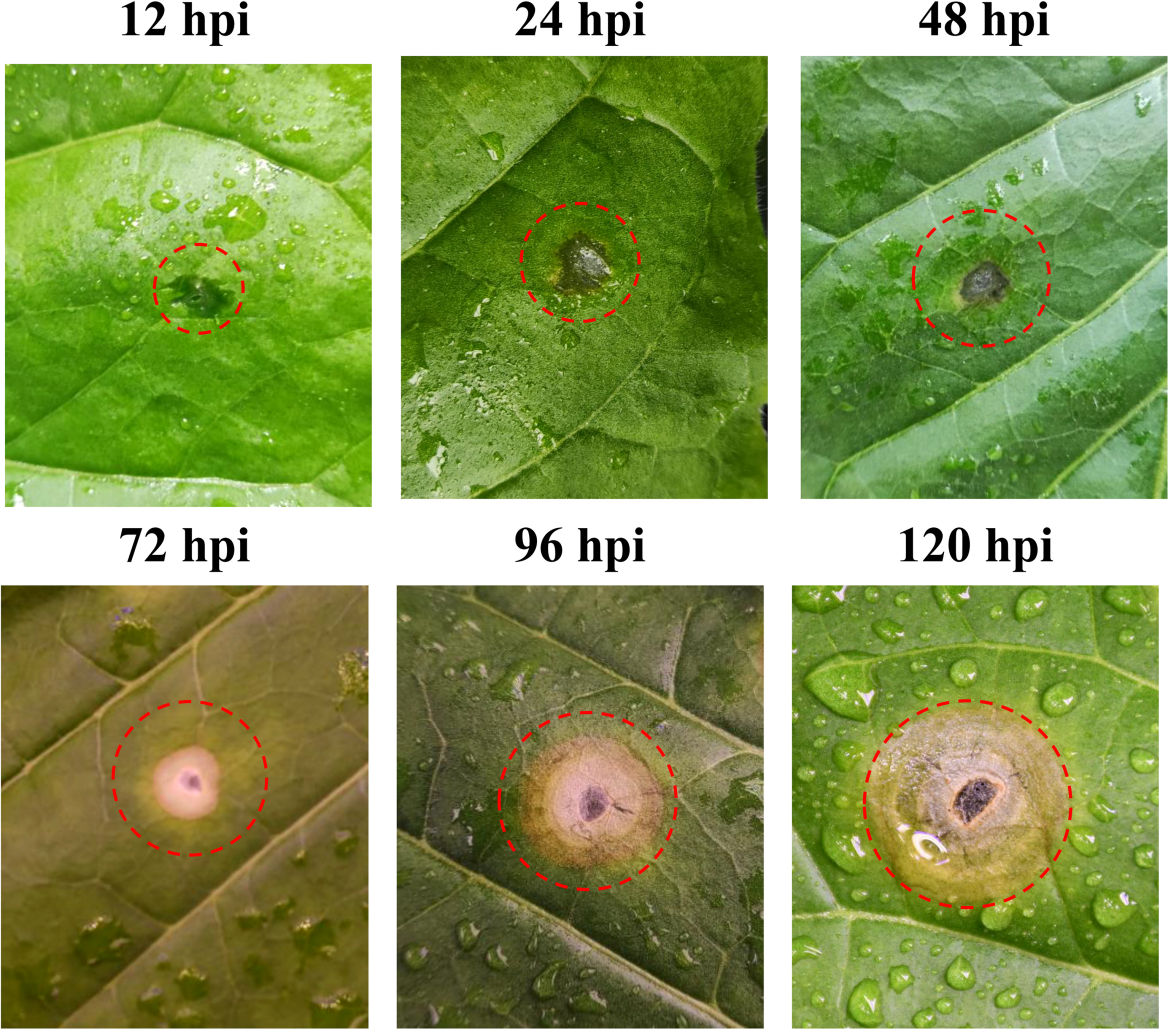
Disease symptoms of tobacco plants after *Rhizoctonia solani* inoculation.

The tobacco leaf symptoms noted after *R. solani* AG3 inoculation are shown in **Figure 1**. At 12 hpi, no obvious change was noted at the inoculation site. At 24 hpi, the wounds gradually expanded and started turning yellow at their edges. At 48 hpi, the inoculation site showed obvious yellowing, and the abaxial side exhibited occasional aerial mycelia. At 72 hpi, the inoculation site developed water-soaked circular or elliptical spots and the lesion site exhibited remarkable chlorosis. The color of the lesion gradually deepened, and it showed two-tiered brown and gray concentric rings at 96 hpi. At 120 hpi, the lesion eventually developed three layers of concentric whorls and showed partial necrosis.

### 3.2 RNA-seq findings

To investigate the changes in the tobacco transcript levels at different stages of infection by *R. solani*, we used high-throughput sequencing technology to assess gene expression in tobacco after inoculation. Transcriptomic analysis of the inoculation experiment results involved analysis at seven time points, with three biological repeats at each time point; thus, a total of 21 samples were tested. The raw RNA-seq data for the 21 tobacco samples were submitted to the NCBI database under accession number SRP400587: SRR21777789 - SRR21777809. After the filtration process, a total of 215.32 Gb clean data were acquired from 21 samples; the total clean reads of each sample varied from 49.85 to 78.58 M. The Q20 and Q30 values for each sample were greater than 99% and 95%, respectively (**Supplementary Table S1**).

### 3.3 Differential gene expression analysis

**Figure 2:**
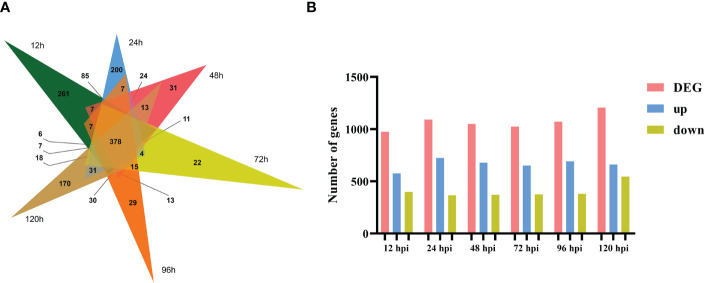
Number **(A)** and Venn diagram **(B)** of total DEGs at 12, 24, 48, 72, 96, and 120 hpi.

To identify which gene expression pattern had changed and to determine the time points at which these alterations occurred, we investigated the number of DEGs at various time points (12, 24, 48, 72, 96, and 120 h) after the tobacco plant was inoculated with *R. solani* AG3 (**Figure 2A**). The lowest number of DEGs (974) was found at 12 hpi; 576 transcripts were differentially induced, while 398 genes were differentially reduced. The highest number of DEGs (1205) was identified at 120 hpi; 661 genes were found to be differentially induced, while 544 were differentially reduced. A total of 1090 DEGs (723 upregulated and 367 downregulated) were identified at 24 h, 1024 DEGs (650 upregulated and 374 downregulated) at 36 h, and 1071 DEGs (690 upregulated and 381 downregulated) at 96 hpi. Among these DEGs, 378 were continuously expressed at all time points after inoculation (**Figure 2B**).

### 3.4 GO and KEGG analyses of DEGs

**Figure 3:**
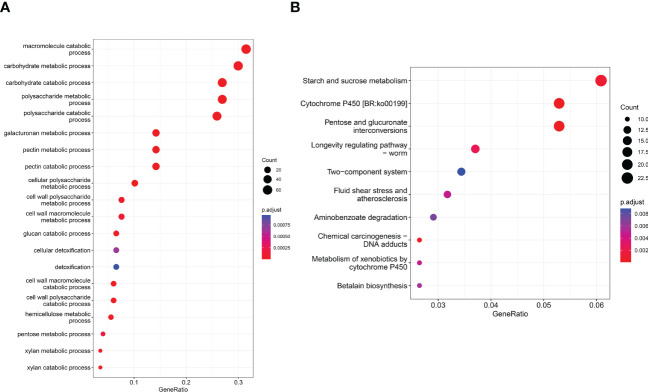
GO term distribution **(A)** and KEGG pathway **(B)** in relation to *R. solani*–tobacco interaction.

Queries were run against the GO database for each DEG in order to categorize and annotate the possible functions of these DEGs at different infection stages (**Figure 3A**). GO enrichment analysis showed that the common DEGs were significantly enriched in metabolism and biosynthesis categories, included “carbohydrate metabolic process,” “polysaccharide metabolic process,” and “amide biosynthetic process.” GO enrichment analysis showed that the GO terms exhibited distinct expression patterns in the different infection stages, and common DEGs were significantly enriched in metabolism and biosynthesis categories. The number of DEGs associated with “pectin catabolic process” gradually increased from 12 to 48 hpi and reached the highest level at 72 hpi; subsequently, slow decline was noted at 96 and 120 hpi but the number remained higher than that at 24 hpi. In addition, the cell wall macromolecule catabolic process and cell wall polysaccharide catabolic process began at 12 hpi and peaked at 48 hpi, while the cell wall polysaccharide metabolic process and macromolecule metabolic process began at 48 hpi and peaked at 96 hpi. The findings suggest that these time points were critical for successful *R. solani* infestation. KEGG enrichment analysis was performed to further investigate the specific *R. solani* pathways involved during plant infection (**Figure 3B**). The findings showed that “pentose and glucuronate interconversions,” “starch and sucrose metabolism,” and “cytochrome P450” were the common significantly enriched pathways during the process of *R. solani* infection. The complete GO and KEGG results are shown in **Supplementary Tables S3**, **S4**.

### 3.5 Classification of genes on the basis of their functions

**Figure 4:**
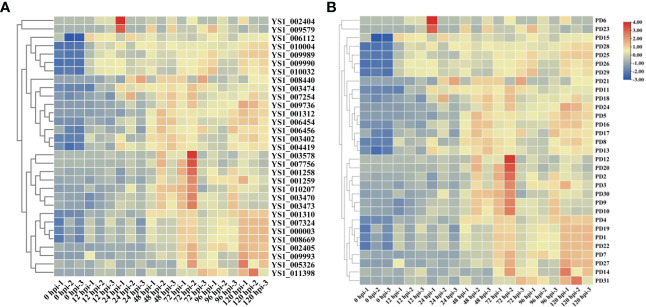
Heat maps showing the expression of cytochrome P450 **(A)** and pectin-degrading enzymes in *R. solani* **(B)**. The values shown with the bars at the right indicate the FPKM of PD genes at various time points.

**Figure 5:**
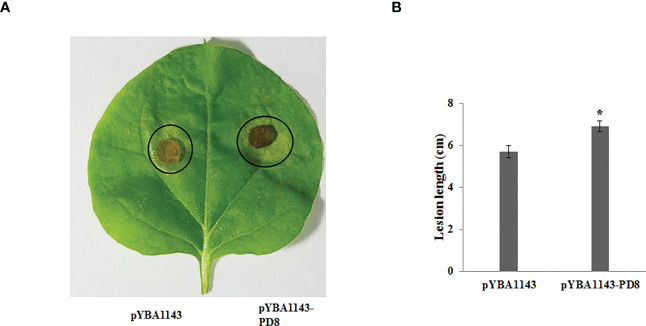
Over-expressing *PD8* lead to enhanced susceptibility to *R. solani*. **(A)** The disease phenotypes **(B)** The lesion diameter of leaves infected by *R. solani* at 2 dpi.

On the basis of the GO and KEGG analyses, we extracted expression data for pectin-degrading (PD) and cytochrome P450 enzymes, considering their key roles in pathogenesis. We found 34 genes potentially encoding cytochrome P450 enzymes exhibited two synchronous but diametrically opposite expression patterns at different time points. These results indicate that the cytochrome P450 gene family might competitively antagonize the functional response at different infection stages (**Figure 4A**). The expression of 31 PD enzyme–encoding genes was analyzed and showed a consistent pattern throughout infection. The expression abundance of the entire PD gene family increased to different degrees, suggesting that PD enzymes play a key role during plant infection (**Figure 4B**). To determine whether PD can enhance the pathogenicity of *R. solani*, PD8 was transiently overexpressed in *N. benthamiana*. We found there were significantly big lesions in PD8–overexpression plants leaves infected by *R. solani* than wild-type (**Figure 5A, B**).

### 3.5 ROS scavenging genes in *R. solani*

**Figure 6:**
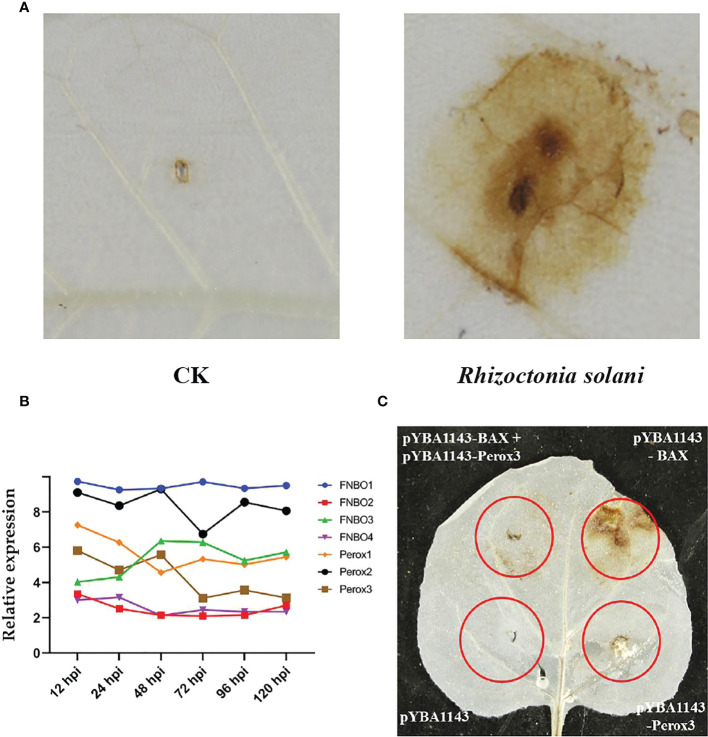
ROS production and scavenging. **(A)** Accumulation of ROS of tobacco plants after *R. solani* inoculation at 72 hpi. CK indicates non-inoculated plants. The yellow regions represent the the accumulated of ROS. **(B)** The expression of *FNBO* and *Perox* genes in *R. solani*. X-axis: 12–120 h: *R. solani*–inoculated tobacco harvested at various time points. Y-axis: Relative expression levels of select genes. **(C)** Analysis of ROS scavenging mediated by Perox3. pYBA1143: empty vector, pYBA1143-Perox3 and BAX: apoptosis regulator. The yellow regions represent the accumulated of ROS.

In addition, we found that reactive oxygen species (ROS) are produced in large quantities in tobacco leaves during *R. solani* infection (**Figure 6A**). Correspondingly, *R. solani* express dedicated detoxification mechanisms in order to counteract host ROS and survive oxidative stress. To elucidate the mechanism of detoxification of H_2_O_2_, we analyzed the relative expression of *FAD/NAD binding oxidoreductase* (*FNBO*) and *peroxidase* (*Perox*) gene family, which play an important role in reducing oxidative damage. Our results showed that four *FNBO* genes and three *Perox* genes have sustained upregulation at different infection stages (**Figure 6B**). Next, we performed a ROS stain experiment by co-injecting Perox3 and apoptosis regulator BAX. The result showed that none of pYBA1143 empty vector and pYBA1143-Perox3 were able to produce ROS, and apoptosis regulator BAX can induce the production of large amounts of ROS. In contrast, co-injection of Perox3 and apoptosis regulator BAX resulted in a significant decrease of ROS production and reveal that Perox3 has a great capacity for ROS scavenging (**Figure 6C**).

### 3.7 Protein-protein interaction network prediction

**Figure 7:**
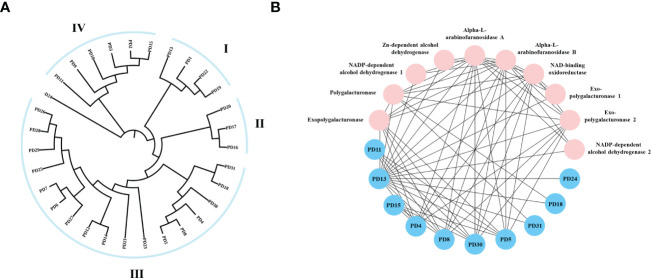
Phylogenetic tree and interaction network of pectin degrading enzymes genes. **(A)** The phylogenetic tree was generated from PD amino sequences using RAxML software by the Maximum Likelihood method with 500 bootstrap replicates. **(B)** Nodes represent proteins, and edges indicate protein-protein interaction. Edges colors indicate the type of evidence for interaction. Asterisk (*) represents significant difference (P < 0.05).

To investigate the evolutionary relationship of *R. solani* PD members, 31 aligned PD protein sequences from *R. solani* were used to construct a phylogenetic tree using RAxML software and the Maximum Likelihood method (**Figure 7A**). The evolutionary tree indicated that the PD family classified into four subfamilies (Named I to IV). The subfamilies with the most members were subclass III (18) and those with the fewest members were subfamilies II (3) whereas PD24 form a separate clade. To further disclose the potential interactions between pectin degrading enzymes proteins, the pectin degrading enzymes protein family interaction network was generated based on the String server (**Figure 7B**). As a result, 32 nodes and 70 edges were obtained in the protein-protein interaction network. PD13 was predicted to interact with 18 pectin degrading enzymes proteins and other enzymes proteins and PD4, PD5, PD8, PD30 were predicted to interact with eight proteins. These results suggested that *PD* were the key pathogenicity-related genes in *R. solani*.

### 3.7 qPCR validation

**Figure 8:**
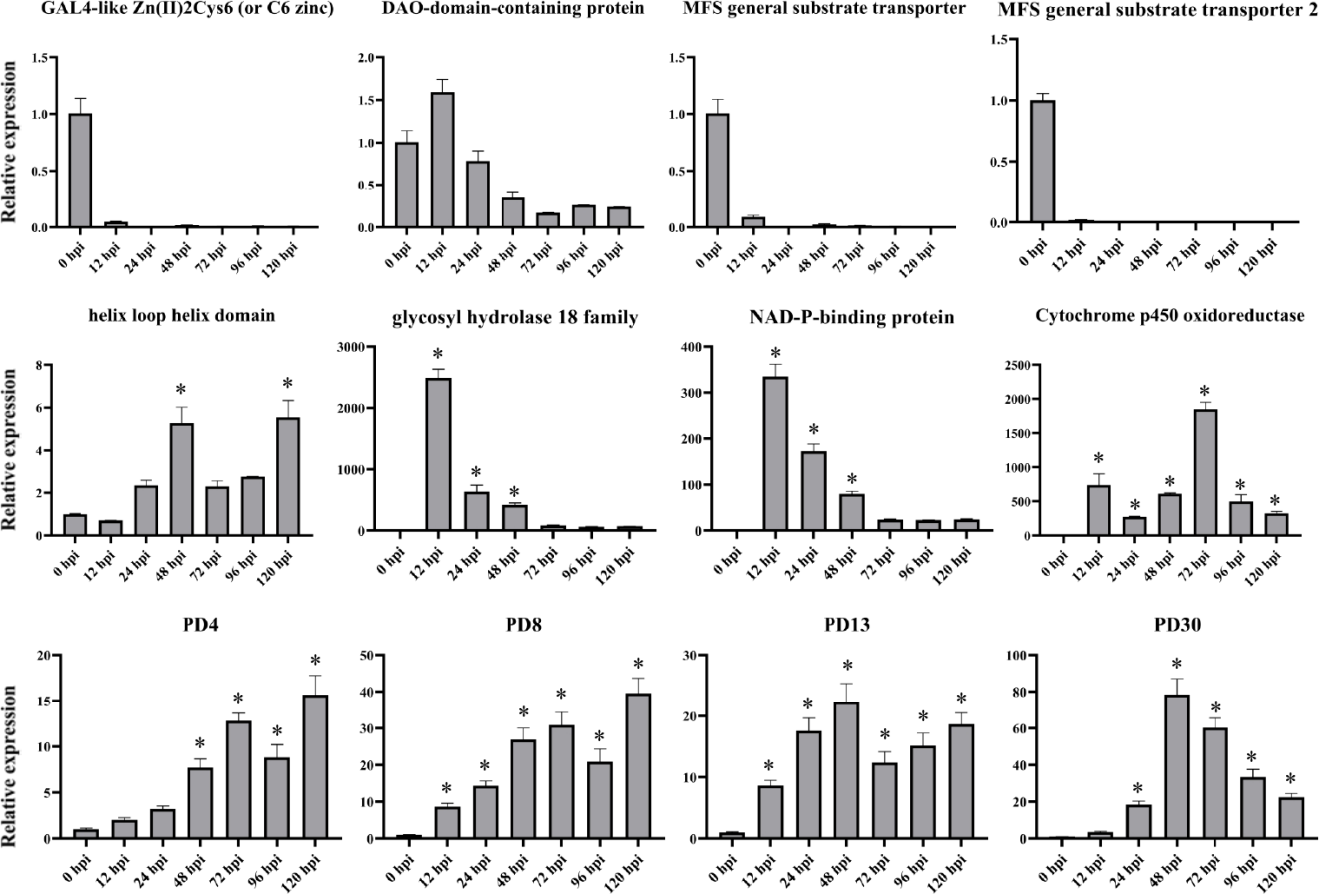
qPCR expression analyses of select genes at various time points post inoculation. X-axis: 12–120 h: *R. solani*–inoculated tobacco harvested at various time points. Y-axis: Relative expression levels of select genes in *R. solani* grown in PDA. Error bars indicate the mean of three biological replicates. Asterisk (*) represents significant difference (P < 0.05).

To verify the RNA-seq results, 12 genes were chosen for qPCR on the basis of their RNA-seq results to further examine their expression patterns (**Figure 8**). These genes are mainly involved in ROS responses and plant defense responses (Sasaki et al., 2002). This analysis included genes that were significantly up- or downregulated in the infected samples at various time points. The expression patterns of these selected genes were validated using qPCR, and the relative fragments per kilobase million (FPKM) transcript levels from the transcriptome exhibited strong correlation, indicating that the RNA-seq results were reliable. Furthermore, we note that the expression patterns of PD4 and PD8 were in good agreement, though the degree of expression varied, which indicated that they may be participated in similar regulation mechanisms as well as similar biological functions.

## 4 Discussion

The utilization of RNA-seq innovations has provided substantial insights into plant–pathogen interactions. However, researchers have only relatively recently started investigating the dynamic transcriptome-wide response of tobacco pathogens, such as *R. solani*, during infection of host plants. Some aspects related to infection mechanism of *R. solani* remain unclear. In the current study, we aimed to provide insights into *R. solani* gene expression during infection. To the best of our knowledge, this is the first RNA-seq–based comprehensive transcriptomic study to characterize the interaction between *R. solani* AG3 and tobacco at the molecular level.

Pectin is a major structurally complex component of the plant cell wall (Du et al., 2022) and is the predominant contributor to host immunity (Molina et al., 2021). The host cell wall is depolymerized by the pectin lyase or pectin esterase of the pathogen and is subsequently utilized as the main carbon source for pathogen growth and metabolism. These enzymes also serve as virulence factors in phytopathogenic microorganisms (Pan et al., 2018). The functional genes encoding these enzymes are therefore potential targets for RNAi- or genome editing–based development of plant disease resistance. *Perox* and *FNBO* gene family is the critical component of the plant pathogens antioxidant defense system, which may contribute to scavenge the production of ROS for host defense and increase resistance to oxidative stress (Choo et al., 2023). In this study, we found that *Perox* and *FNBO* gene family showed high expression level in different infection stages and many gene family members (*FNBO3*, *Perox2*, *Perox3*) showed significantly increased at 48 dpi. We speculate that *Perox* and *FNBO* gene family was integral to *R. solani* maintain a high level in detoxifying the harmful effects of ROS generated from host defense responses.

Only a few studies have focused on the role of PD genes during *R. solani* infection and disease progression. In the current study, 31 PD-encoding genes and their expression patterns were identified and analyzed on the basis of transcriptomic data. We found that some specific members of the PD enzyme gene families were preferentially recruited at different infection stages for pectin depolymerization. This preferential recruitment might also be a coping strategy for pathogens in order to depolymerize the diverse range of polysaccharides present in the cell wall, which consists of 17 diverse sugar monomers linked by more than 20 different glycosidic bonds (Guo et al., 2022). Most PD enzyme genes were strongly induced at 72 hpi, indicating that 72 hpi was the key time point for disease occurrence; this finding matches those of previous study describing the molecular events occurring during infection by the rice SBD pathogen *R. solani* AG1-IA (Rao et al., 2020). Significant induction of Glycosyl hydrolases family (PD1, PD7, PD14, PD19, PD22, PD27, and PD31) was observed at multiple time points, indicating that these are stage-preferential genes that are potential candidates for molecular validation studies on determining their respective roles in *R. solani* infection. PPI network analysis revealed the identity of several functional partners among PD members and PD members is likely to regulate the *R. solani* infection together with the interacting genes. Based on the phylogenetic tree and ppi network, the four modules (PD4, PD5, PD8, PD30) within the same branch from same subfamily and interact with Exo-polygalacturonase, NAD-binding oxidoreductase, Alpha-L-arabinofuranosidase, and Exopolygalacturonase. Therefore, we speculated that these four PD genes had strongly similar biological functions.

Cytochrome P450 enzymes exert effects *via* multiple pathways during the physiological and biochemical processes of the pathogen, especially by catalyzing biotransformation reactions and *via* metabolic clearance of chemical pesticides or host-defensive compounds, which contribute to pathogen survival in hostile surroundings (Oliw, 2021). The cytochrome P450 gene expression profiles noted at various time points post-infection suggested that early- or late-response genes play multiple roles during *R. solani* pathogenesis. The 21 upregulated cytochrome P450 genes in *R. solani* could interfere with the transcription of pathogen-induced defense genes in host plants, thereby inhibiting or limiting host immune responses (Črešnar and Petrič, 2011).

## 5 Conclusions

Our study provides comprehensive RNA-seq analysis of infection with *R. solani* AG3, the causal agent of target spot disease, at seven different time points during tobacco infection. The differential expression of genes in *R. solani* at different infection stages provides several important insights into defense and attack mechanism activation. Overall, this study enhances current knowledge regarding the molecular mechanisms underlying the response of tobacco to *R. solani* AG3 infection and lays the foundation for identifying candidate genes participating in target spot disease resistance in tobacco. The candidate defense- and attack-associated genes identified in this study might provide a basis for future identification of fungal pathogenicity–related genes and also provide a foundation for targeted control methods and novel strategies for developing target spot disease–resistant tobacco lines.

## Data availability statement

All raw sequencing data has been deposited into the NCBI database under accession number SRP400587: SRR21777789 - SRR21777809.

## Author contributions

NS and QT are responsible for study design and manuscript writing. QM, TL, ZX, YX, YW and LL are responsible for data analysis and integrity of research. WP, and BW conceived, designed and edited the manuscript. TL, QT and NS did review and final approval of manuscript. All authors contributed to the article and approved the submitted version.

## Supplementary material

Supplementary Table 1 Summary of RNA-seq results.

Supplementary Table 3 GO term enrichment analysis of DEGs (12, 24, 48, 72, 96, and 120 hpi).

Supplementary Table 4 KEGG pathway analysis of DEGs (12, 24, 48, 72, 96, and 120 hpi).
